# Correction: Optimizing accuracy and efficacy in data-driven materials discovery for the solar production of hydrogen

**DOI:** 10.1039/d2ee90016e

**Published:** 2022-03-17

**Authors:** Yihuang Xiong, Quinn T. Campbell, Julian Fanghanel, Catherine K. Badding, Huaiyu Wang, Nicole E. Kirchner-Hall, Monica J. Theibault, Iurii Timrov, Jared S. Mondschein, Kriti Seth, Rowan R. Katzbaer, Andrés Molina Villarino, Betül Pamuk, Megan E. Penrod, Mohammed M. Khan, Tiffany Rivera, Nathan C. Smith, Xavier Quintana, Paul Orbe, Craig J. Fennie, Senorpe Asem-Hiablie, James L. Young, Todd G. Deutsch, Matteo Cococcioni, Venkatraman Gopalan, Héctor D. Abruña, Raymond E. Schaak, Ismaila Dabo

**Affiliations:** Department of Materials Science and Engineering, and Materials Research Institute, The Pennsylvania State University, University Park PA USA yihuangxiong@psu.edu; Sandia National Laboratories Albuquerque NM USA; Department of Chemistry and Materials Research Institute, The Pennsylvania State University, University Park PA USA; Department of Chemistry and Chemical Biology, Cornell University Ithaca NY USA; Theory and Simulation of Materials (THEOS) and National Centre for Computational Design and Discovery of Novel Materials (MARVEL), École Polytechnique Fédérale de Lausanne CH-1015 Lausanne Switzerland; School of Applied and Engineering Physics, Cornell University Ithaca NY USA; Department of Materials Science and Engineering, Northwestern University Evanston IL USA; Institutes of Energy and the Environment, The Pennsylvania State University, University Park PA USA; National Renewable Energy Laboratory Golden CO USA; Department of Physics, University of Pavia Pavia Italy

## Abstract

Correction for ‘Optimizing accuracy and efficacy in data-driven materials discovery for the solar production of hydrogen’ by Yihuang Xiong *et al.*, *Energy Environ. Sci.*, 2021, **14**, 2335–2348; DOI: 10.1039/D0EE02984J.

The following funding source was missing from the Acknowledgments section:

The National Science Foundation through the Research Experiences for Undergraduates and Research Experiences for Teachers in Nanoscale Physics and Materials at Pennsylvania State University under Grant No., DMR142062 and DMR2011839.

The Royal Society of Chemistry apologises for these errors and any consequent inconvenience to authors and readers.

## Supplementary Material

